# PTH Reloaded: A New Evolutionary Perspective

**DOI:** 10.3389/fphys.2017.00776

**Published:** 2017-10-09

**Authors:** Paula Suarez-Bregua, Laura Cal, Cristian Cañestro, Josep Rotllant

**Affiliations:** ^1^Institute of Marine Research (IIM-CSIC), Vigo, Spain; ^2^Departament de Genètica, Microbiologia i Estadística, IRBio, Universitat de Barcelona, Barcelona, Spain

**Keywords:** PTH family, GPCR, mineral balance, bone homeostasis, Pth4, fish, ohnologs, evolution

## Abstract

The parathyroid hormone (PTH) family is a group of structurally-related secreted peptides involved in bone mineral homeostasis and multitude of developmental processes in vertebrates. These peptides mediate actions through PTH receptors (PTHRs), which belong to the transmembrane G protein-coupled receptor group. To date, genes encoding for PTH and PTHR have only been identified in chordates, suggesting that this signaling pathway may be an evolutionary innovation of our phylum. In vertebrates, we found up to six PTH and three PTHR different paralogs, varying in number between mammals and teleost fishes due to the different rounds of whole-genome duplication and specific gene losses suffered between the two groups of animals. The diversification of the PTH gene family has been accompanied by both functional divergence and convergence, making sometimes difficult the comparison between PTH peptides of teleosts and mammals. Here, we review the roles of all Pth peptides in fishes, and based on the evolutionary history of PTH paralogs, we propose a new and simple nomenclature from PTH1 to PTH4. Moreover, the recent characterization of the Pth4 in zebrafish allows us to consider the prominent role of the brain-to-bone signaling pathway in the regulation of bone development and homeostasis. Finally, comparison between PTH peptides of fish and mammals allows us to discuss an evolutionary model for PTH functions related to bone mineral balance during the vertebrate transition from an aquatic to a terrestrial environment.

## Introduction

One of the innovative features that characterize vertebrates is the bone, a stiff tissue with high capacity for regeneration. The evolution of bone has been accompanied by the development of a hormonal system that allows a precise control of bone mineral metabolism. Within this system, the parathyroid hormone (PTH) family of peptides plays key roles in the homeostasis of calcium-phosphate that act as regulators in numerous biological processes, such as the formation of hydroxyapatite crystals for bone mineralization (Potts, 2005; McCauley and Martin, 2012).

In mammals, there are three known genes that code for PTH peptides, which classically has been termed as “parathyroid hormone” (PTH), “parathyroid hormone related protein or PTH-like hormone” (PTHrP, PTHLH), and “tuberoinfundibular peptide of 39” (TIP39, PTH2). Moreover, mammals have two genes that code for PTHRs within the class B G protein-coupled receptors (GPCRs) (Venkatakrishnan et al., 2013), which has been termed as “parathyroid hormone type I or II receptor” (PTH1R, PTH2R). In mammals, PTH is secreted by the parathyroid gland (PTG), which functions as the major endocrine regulator of the calcium-phosphate metabolism. PTH can directly acts in bone and kidney, and indirectly in intestine interacting with local and systemic factors to restore normal serum levels in a feedback-loop (Brown, 1993). PTHLH is not secreted by the PTG, but it has a widespread spatial distribution, mainly participating in the embryonic development of the skeleton in an autocrine/paracrine fashion and promoting the calcium mobilization as an endocrine factor during gestation and lactation (Neville et al., 2002; VanHouten et al., 2004; Kronenberg, 2006). Additionally, PTH and PTHLH have a common and paradoxical effect in bone. Both promote bone resorption or formation through PTH1R depending on whether the dose is continuous or intermittent, respectively (Silva et al., 2011). In fact, human PTH(1-34) and PTH(1-84) are the only approved anabolic agents up to date for the treatment of osteoporosis (Moen and Scott, 2006; Cosman et al., 2017). Thus, one of the current hottest topics in the field of bone research is to develop novel PTH analogs that can be used as pharmaceutical drugs that promote bone formation or inhibit resorption. TIP39 is the peptide that shows the smallest amino acid sequence similarity to PTH and PTHLH, which despite has been described to be able to bind PTHR2, and potentially compete with PTH, its role on bone metabolism remains unclear (Usdin et al., 1999).

In contrast to mammals, the PTH family in fishes has acquired a higher complexity, consisting on at least six genes resulting from the extra-round of whole-genome duplication (WGD) occurred at the base of the teleost lineage: two Pth paralogs (Ptha/Pthb), two Pthlh (Pthlha/Pthlhb), Tip39(Pth2) and a new PTH-like peptide named Pth4 (Guerreiro et al., 2007; Suarez-Bregua et al., 2017). In this review, we examine the roles of all PTH peptides in fishes, and based on the evolutionary origin of PTH paralogs, we propose a new and simple nomenclature from PTH1 to PTH4. Moreover, we highlight how the recent discovery and characterization of a Pth4 in fishes, a paralog absent in eutherians, including humans, reinforces the key role that the brain-to-bone signaling pathway has on the regulation of bone homeostasis. Finally, comparison between the features of PTH peptides of fish and mammals allows us to discuss a plausible scenario for the evolution of PTH functions related to bone mineral balance during the vertebrate transition from an aquatic to a terrestrial environment.

## Evolutionary-based novel PTH family nomenclature

**Figure 1:**
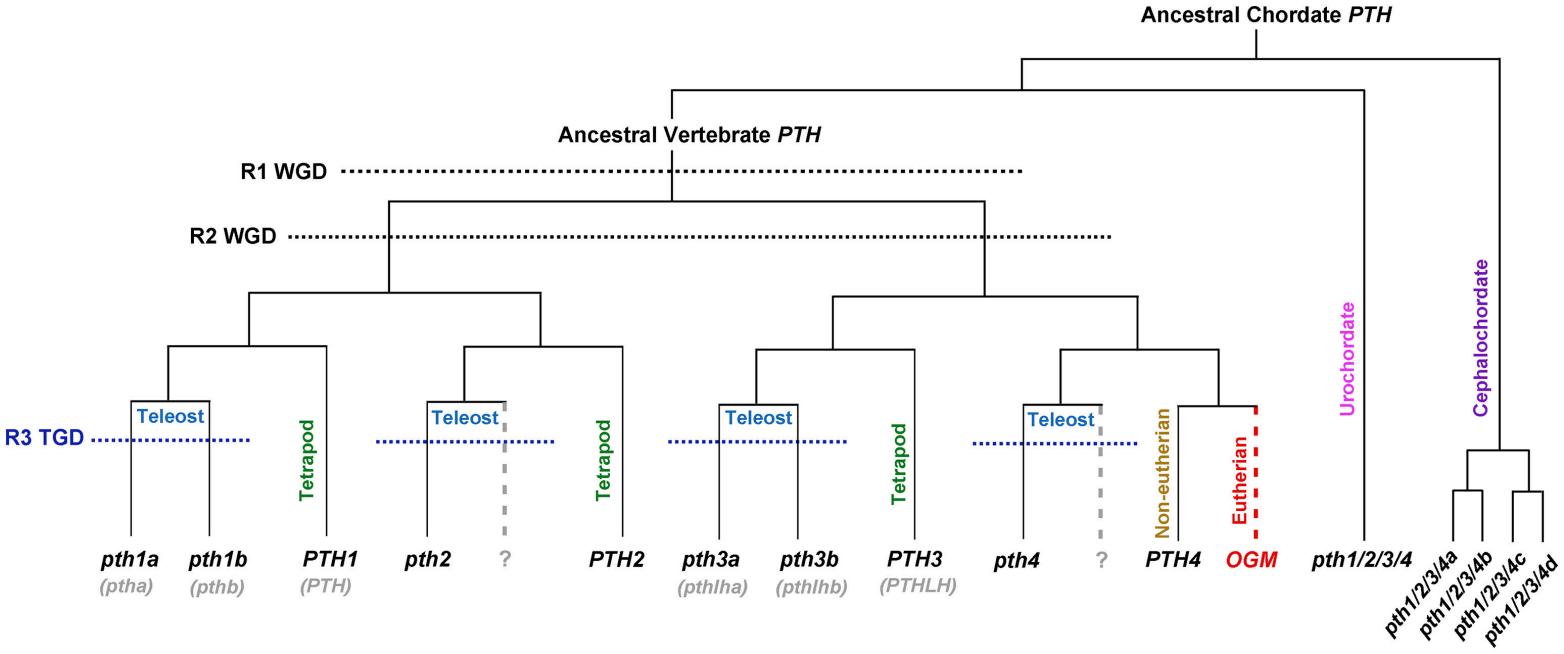
Evolutionary model and revised nomenclature proposed for *PTH* family in vertebrates. *PTH* family members (*PTH1, PTH2, PTH3*, and *PTH4*) are ohnologs that arose from a *PTH* ancestor because of two rounds of whole genome duplications (R1 WGD and R2 WGD) that occurred at the base of the vertebrate radiation. An additional third round of genome duplication was specific to the teleost lineage (R3 TGD) and gave rise to duplicated *pth1a/pth1b* and *pth3a/pth3b*. The hypothetical loss of a *pth2* and *pth4* co-orthologs in fish is denoted by gray dashed line. The *PTH4* Ohnolog-gone-missing (OGM), absent in eutherian vertebrates, is indicated by red dashed line.

The fact that genes encoding for PTH peptides and PTHRs have been found so far only in amphioxus, urochordates and vertebrates suggests that the PTH signaling pathway is an evolutionary innovation of our own phylum, the chordates (Mirabeau and Joly, 2013). Phylogenetic inferences and analyses of conserved synteny between the genomes of different vertebrates have helped to elucidate the evolutionary origin of each member of the tangled PTH repertoire in fish and mammals (Figure 1; Yan et al., 2012; Suarez-Bregua et al., 2017). The finding of four PTH paralogous genes in four genomic regions with conserved synteny strongly supports that their origin is due to the two rounds (R1/R2) of WGD that occurred during early vertebrate evolution (Dehal and Boore, 2005; Putnam et al., 2008), reviewed in Cañestro (2012). Analyses of conserved synteny have also revealed that the absence of PTH4 in eutherians is due to an ancestral gene loss that occurred after the eutherian-metatherian split (Figure 1). These main four PTH paralogs of vertebrates are therefore ohnologs, term coined honoring Susumo Ohno that refers to a special type of paralogs originated by WGD (Wolfe, 2000). PTH4 should be considered an eutherian Ohnolog-gone-missing (PTH4-OGM)-concept adopted to describe ohnologs that have been lost in specific taxa (Postlethwait, 2007), which today should have been present in a region of the human chromosome 6 where some genes still show conserved synteny with neighbor genes present in the regions of the other PTH ohnologs (Suarez-Bregua et al., 2017). In the case of teleosts, phylogenetic inferences and analyses of conserved synteny show that additional Pth paralogs (a/b, Figure 1) have originated by the extra-round of teleost genome duplication (TGD) (Braasch and Postlethwait, 2012; Suarez-Bregua et al., 2017). Based on the evolutionary history of the PTH family, and to simplify the complexity of current Pth names, we propose a new nomenclature in which main PTH ohnologs are numbered from 1 to 4, and each extra fish paralog due to TGD are named with a or b (Figure 1; Suarez-Bregua et al., 2017). In the case of Pth of non-vertebrate chordates (cephalocordate and urochordate species) Pth genes should be named Pth1/2/3/4 (followed with a/b/c…in case of extra taxon-specific paralogs), since all of them are equally co-orthologs to any vertebrate PTH gene.

Similarly, conserved synteny studies revealed that PTHRs evolved through genome duplication and gene loss. It has been postulated that the ancestor of teleosts and tetrapods had three PTHRs and a putative PTH4R was lost after R2 of duplication (Bhattacharya et al., 2011). However, the identification of three potential PTHRs in non-chordates suggests that receptors arose before than PTH ligands and evolved under different evolutionary pressure (Pinheiro et al., 2012).

## PTH family in fish

Despite the absence of PTG in fish, historically it has been known that fish could respond to changes in mineral levels in serum, and consequently it was presumed that fish should also have a hormonal system able to regulate mineral homeostasis, likely related to bone formation. Next, we will review the Pth family in fish following the historical order in which each peptide was discovered.

## Pth3 (Pthlh)

Pthlh was the first PTH peptide isolated in fish and the best functionally characterized. Initial studies by Parsons et al. (1978) using mammalian PTH antiserum, had shown evidence of a PTH-like factor in cod and eel pituitaries with hypercalcemic effects in rainbow trout. In 1991, Fraser and colleagues found Pth3 in the pituitary of coho salmon and in 1993 Danks et al. identified this factor in plasma and pituitary of seabream using an antiserum against human PTH3(1–16) (Fraser et al., 1991; Danks et al., 1993). Subsequently, *pth3* was isolated in diverse fish tissues such as gills, operculum, kidney, pituitary, brain, saccus vasculosus, muscle, skin, spleen, liver and intestine (reviewed by Abbink and Flik, 2007). In pufferfish and zebrafish two *pth3* were identified and cloned, *pth3a/pth3b* (Power et al., 2000; Canario et al., 2006; Yan et al., 2012), however only *pth3a* has been identified in seabream (Flanagan et al., 2000). Recent studies have outlined the importance of Pth3 in fish ion balance (Gregório et al., 2014). The increase of circulating Pth3 in seabream stimulates the calcium uptake through gills and intestine (Guerreiro et al., 2001; Abbink et al., 2006; Fuentes and Figueiredo, 2006). Likewise, Pth3 also seems to be involved in other functions such as the estradiol-induced mineral mobilization during vitellogenesis (Fuentes et al., 2007), skeletal mineralization by reducing the expression of the osteonectin gene (Redruello et al., 2005), and in calcium mobilization from scales through the increase of osteoclast activity (Rotllant et al., 2005). Additionally, a study has demonstrated that intermittent administration of Pth3 in seabream caused a modification of the bone proteome suggesting an anabolic response on the skeleton (Anjos et al., 2013). Besides Pth3 also stimulates renal phosphate secretion in winter flounder (Guerreiro et al., 2010). Regarding to the involvement in embryonic development of the skeleton, zebrafish Pth3 seems to have a conserved role to that of mammals. It was shown that Pth3 co-orthologs have different craniofacial expression patterns and loss-of-function studies showed that they play different roles in skeletogenesis through interactions with their upstream regulator Sox9 and downstream target Runx2. A hypothesis have been proposed respect to a possible dual role of Pth3a/Pth3b as a paracrine hormone for chondrogenesis/osteogenesis and as a circulating hormone for serum calcium-phosphate homeostasis (Yan et al., 2012).

## Pth1 (Pth)

After the isolation of Pthlh genes in teleosts, many efforts were made to find Pth homologs (Flanagan et al., 2000; Power et al., 2000). To date, two types of Pth has been identified in pufferfish (pPth1a, pPth1b) and in zebrafish zPth1a and zPth1b (previously zPth1, zPth2) (Danks et al., 2003; Gensure et al., 2004; Canario et al., 2006). Spatial expression studies by RT-PCR and *in situ* hybridization (ISH) have shown *zpth1a* and *zpth1b* in similar expression pattern, including cells along the lateral line and notochord and neural tube, with the exception of the central nervous system (CNS) cells, where only *zpth1a* has been detected. On the other hand, immunohistochemistry assays using fugu Pth1a antiserum revealed expression in neuromasts from the lateral line but also in the calcifying jaw of zebrafish, suggesting a possible role in skeletogenesis (Hogan et al., 2005). It has been also shown expression of *pth1a* and *pth1b* in gills of zebrafish and pufferfish (Okabe and Graham, 2004). Knockdown of *pth1a* in zebrafish showed defects in the jaw development and branchial arches, where the expression of a chondrocyte marker (*collagen-2a1a*) was also decreased (Kwong and Perry, 2015). Moreover, it has been pointed out that the tetrapod PTG and the gills of fish are evolutionarily related structures, both arise from endodermal pharyngeal pouches and formed under the control of a crucial regulatory gene, *gcm-2*. Thus, it has been suggested that Pth1 in fish could play a key role in calcium homeostasis similar to mammals (Okabe and Graham, 2004; Canario et al., 2006). Surprisingly, to our knowledge few functional studies have addressed Pth1 roles in bone mineral homeostasis in fish. Suzuki et al. (2011) found that synthetic pPth1a acts on the goldfish scales stimulating osteoclast activity to mobilize calcium through *in vitro*/*in vivo* assays. This hypercalcemic action of pPth1a was not found in seabream scales and neither peptide had any effect in calcium influx in larvae (Canario et al., 2006). However, overexpression and loss-of-function studies have shown that only *zpth1a* increases the calcium uptake and epithelial calcium channel expression in zebrafish (Lin et al., 2014). Also, *zpth1a* expression and ionocytes differentiation were stimulated after acclimation to low calcium water (Kwong and Perry, 2015). Overall, the functions of Pth1s in fish still remains unclear and need to be precisely investigated.

## Pth2 (Tip39)

PTH2, formerly TIP39, was originally purified from bovine hypothalamus and shows low amino acid sequence similarity to PTH1 and PTH3 (Usdin et al., 1999). PTH2 is the endogenous ligand of PTH2R in vertebrates and only a truncated analog in the N-terminal region works as a potent hPTH1R antagonist (Hoare et al., 2000; Jonsson et al., 2001). All avian genomes analyzed lacked a homolog of vertebrate PTH2 while in other vertebrate genomes just a single-gene for PTH2 has been identified. Its expression in thalamic/hypothalamic areas in mammals has suggested regulation of nociception and pain (Dimitrov et al., 2010, 2013), but also of other hypothalamic hormones (Dobolyi et al., 2012). In fish, Pth2 is also specifically expressed in the CNS suggesting a functional conservation between mammals and fishes (Papasani et al., 2004). In contrast to the other Pth family members, Pth2 has not yet been linked to any function related to bone mineral homeostasis.

## Pth4 (Pth-l)

**Figure 2:**
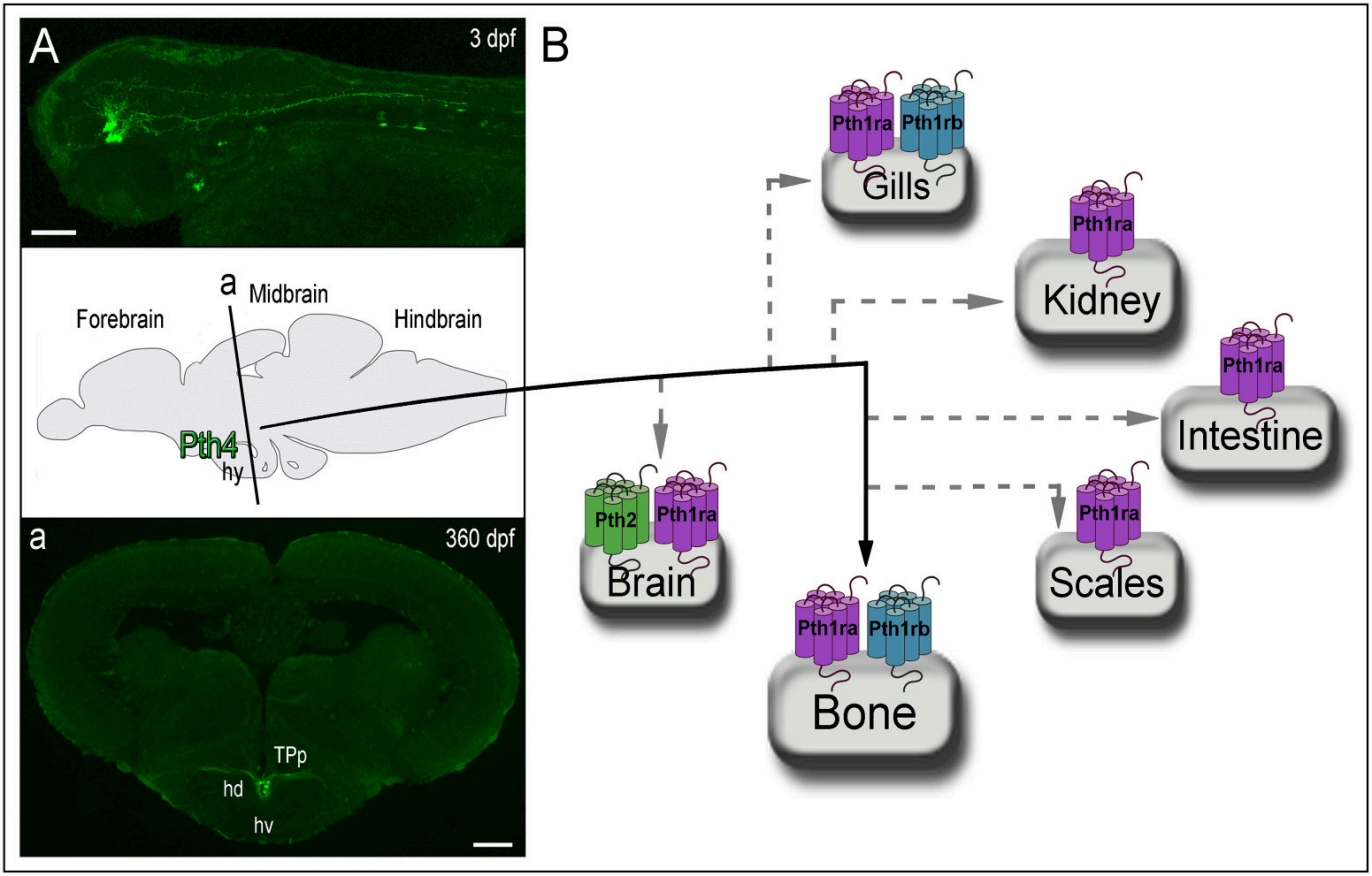
Zebrafish animal model reveals Pth4 acting in a new brain to bone pathway and the possible signaling routes through Pth receptors. **(A)** Left top panel displays pth4:eGFP reporter transgenic fish and a schematic picture of a cross section (denoted by “a”) of adult transgenic zebrafish. (a) Confocal imaging shows Pth4 neuropeptide produced by a specific subset of neurons in the dorsal part of the periventricular hypothalamus and multiple branched projections. **(B)** From the hypothalamus, Pth4 signals bone probably through Pth1ra and/or Pth1rb. Gray dashed lines denote possible signaling pathways via Pth1ra, Pth1rb and Pth2r, which are present in other mineral balance-related organs. Abbreviations: hy, hypothalamus; hd, dorsal hypothalamus; hv, ventral zone of periventricular hypothalamus; TPp, periventricular nucleus of posterior tuberculum. Scale bar: 50 μm **(A)**; 200 μm (a).

The most recent member of the PTH family identified is the Pth4, formerly Pth-l. It was initially identified by *in silico* analysis in pufferfish (Canario et al., 2006) and showed to have intermediate characteristics between Pth1 and Pth3. Preliminary bioactivity studies demonstrated that pufferfish Pth4(1-34) was able to induce a significant *in vivo* stimulation of Ca^2+^ influx in seabream larvae. Due to its effective activity to mobilize calcium, which was greater compared to Pth3(1-34), it was suggested that it might have a PTH equivalent function in fish as in mammals (Canario et al., 2006; Guerreiro et al., 2007). Subsequently, this new PTH ohnolog was identified in chicken and frog, and was also shown to have a role in calcium homeostasis (Pinheiro et al., 2010). But it has not been until 2017 when Suarez-Bregua et al. documented the Pth4 isolation and characterization in zebrafish (Suarez-Bregua et al., 2017). Through comparative analyses of several vertebrate genomes they demonstrated that *pth4* is an ancient PTH lost in eutherian mammals. They suggested that *pth4* was already present in the last common ancestor of Actinopterygii and Sarcopterygii after 1R/2R-WGDs, and although it was conserved across vertebrate evolution, this fourth ohnolog was lost probably after the eutherian-metatherian split (Figure 1). One plausible hypothesis is that chromosomal rearrangements in the mammalian radiation may have contributed to the loss of *PTH4* in eutherians. Similarly, the absence of *pth2* or *pth4* duplicated genes in teleosts after TGD may be due to substantial rearrangements that gave rise to concomitant gene losses. Through ISH experiments and reporter transgenic lines they demonstrated that Pth4 is synthesized by two clusters of hypothalamic neurons with axonal projections to the brainstem and spinal cord (Figure 2), which suggested a systemic role throughout the entire animal. The targeted elimination of these *pth4*-expressing neurons by laser ablation led to abnormal skeletal mineralization during zebrafish development. They also demonstrated that *pth4* expression is directly regulated by Runx2, and that Pth4 could activate downstream signaling mediated by Pthrs (Figure 2). Furthermore, gain-of-function analysis in adult transgenic zebrafish showed that Pth4 acted as a neuropeptide in bone mineral density through the phosphate homeostasis regulation. Overall, their results define a new neural brain-to-bone pathway involving efferent neural signal from hypothalamus to bone receptors controlling bone mineral homeostasis. Although, further investigations are needed to fully understand the Pth4 system, and whether Pth4 mode of action implies an efferent neural signaling via spinal cord and/or if a neuroendocrine pathway would also be feasible.

## PTH family of receptors

To date, three PTHRs have been identified in vertebrates (PTH1R-PTH3R). In mammals, PTH1R is activated by PTH1 and PTH3. It is highly expressed in bone and kidney and regulates extracellular Ca^2+^ homeostasis and bone turnover (Gensure et al., 2005). PTH2R, abundant in brain and pancreas, is essentially activated by PTH2 (John et al., 2002; Papasani et al., 2004). In fish, three receptors have been isolated Pth1r(Pth1ra), Pth2r and Pth3r(Pth1rb) (Rubin and Juppner, 1999) but the affinity, specificity, and the physiological roles are far from defined. Pth1ra is expressed in scales and vertebral bone of seabream (Rotllant et al., 2005; Anjos et al., 2013), in kidney of winter flounder (Guerreiro et al., 2010), and gills, scales, craniofacial bones, intestine, kidney, spine, brain and spinal cord in zebrafish (Kwong and Perry, 2015; Suarez-Bregua et al., 2017). Pth1rb is expressed in vertebral bone and intestine of seabream (Rotllant et al., 2005; Anjos et al., 2013), gills and bone of zebrafish (Kwong and Perry, 2015), and kidney in winter flounder (Guerreiro et al., 2010) suggesting, therefore, a potential role in calcium-phosphate balance in fish similar to Pth1ra. Finally, despite the expression of *pth2r* has been demonstrated in brain, eye, notochord, otic vesicle, pharyngeal arches, ovary and testis in zebrafish (Bhattacharya et al., 2011; Kwong and Perry, 2015), its function remains poorly understood. Despite the phylogenetic distance between fish and mammals, PTH peptides and receptors are able to interact each other and trigger signaling activation which indicates a strong molecular conservation (Rubin and Juppner, 1999; Hoare et al., 2000; Gensure et al., 2004; Rotllant et al., 2005; Suarez-Bregua et al., 2017).

## Evolutionary model for the PTH family members related to the vertebrate transition from aquatic to land environment

While PTH seems the main regulator of bone mineral homeostasis in mammals, Pth4 seems to play the equivalent role in fish (Suarez-Bregua et al., 2017). Phosphate availability in water is scarce compared to calcium and, therefore, a tight phosphate regulation according to body requirements is vital in aquatic vertebrates. Released from the brain, Pth4 acts in the skeleton to maintain bone mineral homeostasis. Pth3 peptides mainly participate in calcium absorption from surrounding waters or mineral mobilization from scales if required as well as in skeletal development. Pth might act in calcium regulation but further investigations would be necessary to accurately clarify those roles. As consequence of evolution from aquatic to terrestrial environment, the calcium uptake from external sources was no longer possible and the appearance of PTG in tetrapods allowed an efficient endocrine control of bone mineral metabolism centralized in PTH. PTH3 would conserve its functions in developmental processes but also in calcium mobilization from skeleton. PTH, structurally more similar to PTH4, would regulate calcium and phosphate balance, while PTH4 would have a certain degree of functional redundancy that facilitated its loss during the evolution of eutherians (Figure 1).

## Conclusion and future perspectives

Although the PTH family has extensively been studied in eutherians, the physiological functions of most fish Pth peptides had not been thoroughly investigated. The recent characterization of new members of the PTH family from non-mammalian species have shown new regulatory pathways of bone homeostasis and revealed a new model for the evolution of the PTH family roles in bone mineral homeostasis in the context of the vertebrate transition from aquatic to terrestrial environments. Therefore, the neural regulation of the bone is an unknown and emergent field of research that is necessary to explore. Further functional analysis will allow characterize the precise modes of action of the recently discovered member Pth4 in the whole fish body. Additionally, future functional studies of Pth4 in basal vertebrates (Lamprey) will help to step forward to decipher the complete evolutionary history of PTH family. Furthermore, we believe that the discovery and characterization of new members of the PTH family from non-mammalian species has the potential to translate to novel therapeutic agents to treat human bone diseases.

## Author contributions

PS-B, LC, CC, and JR wrote and revised the manuscript.

### Conflict of interest statement

The authors declare that the research was conducted in the absence of any commercial or financial relationships that could be construed as a potential conflict of interest.
